# Mass Spectrometry Group Has Mass Appeal

**DOI:** 10.1289/ehp.112-1247668

**Published:** 2004-11

**Authors:** Mary Eubanks

The field of proteomics seeks to define, on a global scale, the levels, activities, regulation, and interaction of proteins in a biological sample. Proteomics is analogous to transcriptomics—the global analysis of mRNA transcripts that arise from the expression of genes in the genome—although the former is considerably more complicated. Whereas the human genome comprises approximately 30,000 genes, there are likely over 100,000 unique proteins in the human proteome due to the multiple ways each gene can be transcribed and translated into proteins by cellular machinery.

Because proteins are fundamental components of all living cells, including enzymes, hormones, and antibodies, they are constantly in flux as the body takes in food, metabolizes it, and stores or burns energy. Furthermore, different proteins are produced and expressed at different developmental stages of an organism’s life cycle, from the moment of conception throughout the aging process. Consequently, proteomics is a dynamic, challenging research area. One of the essential tools being used to meet this challenge is mass spectrometry (MS), which is the focal point of the National Center for Toxicogenomics (NCT) Mass Spectrometry Group.

There are a large number of proteins in a system at any one time, and they are always changing as an organism eats and metabolizes food, exercises, and sleeps. The proteome—all the proteins expressed in a living system—is therefore unique to the cell or tissue under study. The more narrowly one can define where the proteins are localized within the system (such as in a specific tissue or location in a cell), the easier it is to characterize the specific proteins and quantify their varying levels of expression.

Proteomics research at the NCT focuses largely on changes in an organism’s proteome in response to an event such as exposure to an environmental toxicant, in order to advance understanding of how people might respond to chemical exposures in their environment. The Mass Spectrometry Group, led by NIEHS principal investigator Kenneth B. Tomer, employs high-throughput techniques, including MS, to examine hundreds or thousands of protein changes in a large number of samples. Because proteomics techniques produce large amounts of data, sophisticated analysis tools are used to decipher the results and identify key changes in select protein biomarkers that convey valuable information about exposure to harmful chemicals. The MS facility performs analyses for the intramural NIEHS research community, and Tomer also conducts his own collaborative research projects as part of the NCT Proteomics Group.

## Critical Mass

MS enables identification of the composition of a compound based on its mass-to-charge ratio. A sample is ionized, and the charged molecular particles are propelled through an electromagnetic or electric field for separation by differences in mass (molecular weight). A detector records the abundance and mass information for each charged mass and produces a pattern called a mass spectrum. The composition of the sample can be determined based on the mass and relative abundances of ions.

The proteins coming from a specific tissue, cell, or cellular component are separated before they are identified using MS. Separation can be done simply at the whole-protein level using two-dimensional gel electrophoresis; this technique separates molecules by molecular weight and isoelectric point (the pH at which a molecule carries no net electrical charge). The proteins are visualized with various dyes as bands or spots on the gel. The proteins can be more easily identified after the band or spot is cut out, at which point they are digested with enzymes (typically the pancreatic enzyme trypsin) into smaller peptides.

The resulting peptides are identified by “mass fingerprinting,” in which masses of the individual peptides from a gel band are compared with peptide sequences included in the National Center for Biotechnology Information protein database (**http://ncbi.nlm.nih.gov/**). There are publicly available search engines that can match the mass fingerprint against this database for over 1,000 different types of organisms. The search produces a list of possible proteins that might match the mass fingerprint and gives a probability score.

A high probability score indicates a good match between the unknown peptide sequence and a sequence in the database. The lower the score, the less likely it is that the unknown protein matches peptides in the database. For example, if 10 peptides can be identified in a sample and all 10 have a high probability for match accuracy with a particular protein in the database, then there is high confidence that the identification is accurate. However, if the match probabilities are low, the identification cannot be made with any degree of certainty.

A researcher can then go a step further toward identifying the unknown peptides by using an instrument known as a tandem MS to obtain sequence information on their constituent amino acids. This information complements mass assignment and improves identification reliability. This procedure involves digestion of an entire sample with trypsin, followed by separation of the peptides with a high-performance liquid chromatograph and then identification with sequential MS steps. At the second step of MS, the peptide ion is fragmented into smaller amino acids to obtain the mass and sequence information. When sequence information for amino acids correlates well with mass assignment, there is a stronger case for the identity of the protein. Because the result is a probability, additional separation and identification techniques such as antibody arrays and/or Western blotting are employed to provide backup data to corroborate important identifications.

## Cracking a Tough Nut

A particularly challenging area of study within proteomics is post-translational modification of proteins. Post-translational modification is a process by which proteins undergo specific structural changes at certain sites that impart special functions to the protein. The cellular processes involved in post-translational modifications are highly dynamic and very localized, such that modifications can be added or removed very rapidly to a small portion of each protein population as the cell requires. The transient, localized, and proportional nature of post-translational processes makes it difficult to detect this special subset of molecules. For example, says Tomer, phosphorylation is a frequent post-translational modification involved in cell signaling, but even during active signaling, phosphorylation may occur at only one spot on a protein and upon less than 5% of that particular protein population for less than one minute. Yet this type of tiny, rapid modification is vital to cellular function—and crucial to our understanding of toxicity.

Tomer and NCT Proteomics Group toxicologist Alex Merrick addressed the phosphorylation question directly in a model system designed to increase their chances of finding phosphorylations on p53, a key protein controlling cell growth and death. In research published 3 April 2001 in *Biochemistry*, they separated p53 from the proteome of an expression line of Sf9 insect cells in which some cells had been exposed to okadaic acid, a natural phosphatase inhibitor produced by marine algae. Phosphatase inhibition by okadaic acid results in an overall increase in protein phosphorylation and an imbalance in cell signaling that leads to toxicity.

Using MS, Tomer, Merrick, and colleagues identified a number of amino acid sites on the p53 protein that were phosphorylated, and they further discovered that okadaic acid completely phosphorylated one particular site on p53 (serine 315). They speculate that phosphorylation at that site may have particular biological significance for p53, and that studying these processes could improve understanding of the health consequences resulting from phosphorylation of proteins through exposure to environmental contaminants.

## The Power of Comparison

Merrick points to protein profiling as an important use of proteomics in toxicology (toxicoproteomics). Protein profiling, he says, allows a “differential quantification of proteins in one sample that are compared with another sample to see the differences in protein expression.” Comparison of protein differences after exposure to toxicants is one way to find out which proteins respond to the chemicals. Sometimes, these changed proteins lead to unexpected discoveries during the course of toxicoproteomics studies.

For example, it is well recognized that dioxin has major effects on the immune system, but how dioxin mediates these effects is still a mystery as the exact biochemical targets have not been identified. A collaborative study between Tomer, Merrick, and Nigel Walker of the National Toxicology Program, published 15 October 2002 in *Archives of Biochemistry and Biophysics*, led to the discovery of immune-responsive proteins in the endoplasmic reticulum, a region of the cell where they had not been observed before. This is an example of how, in addition to identifying proteins involved in toxicity, proteomics research can be extended into discovery of specific areas within the cell where a toxic action occurs.

The NCT Mass Spectrometry and Proteomics groups, in concert with the NIEHS Micro-array Center, are currently investigating the toxicity of acetaminophen. Although acetaminophen is normally a safe and effective analgesic when used in accordance with the manufacturer’s instructions, cases of overdose or patient susceptibility do occur, and are cause for emergency room visits and concern to public health. Certain populations such as small children, older adults with compromised liver function, and substance abusers are more susceptible to overdose complications. By using discovery genomics and proteomics technologies to profile gene and protein expression in experimental animals, NCT scientists hope to use MS and other technologies to identify biomarkers in the liver and blood that will be informative about the degree of toxicity and prognosis for survival and recovery.

Advances in MS techniques for pro-teome analysis have made this tool an excellent choice for the identification and quantification of proteins and post-translational modification of proteins, with a high level of specificity and sensitivity. Through collaborative research on a variety of intramural projects, NCT researchers are employing MS to shed light on how protein expression and protein–protein interactions are affected by exposure to different environmental toxicants, and are making progress toward development of protein biomarkers that can be used for diagnosing exposure.

## Figures and Tables

**Figure:**
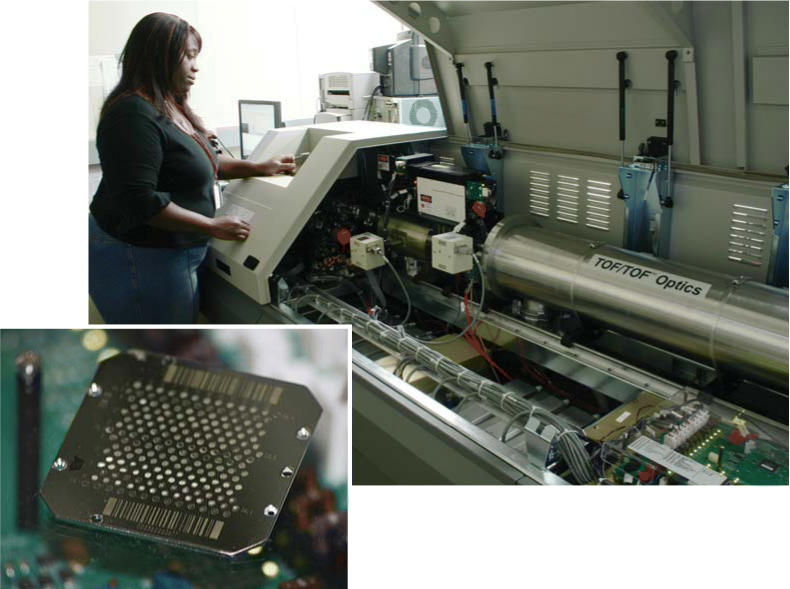
**Keeping an eye on ions.** After proteins are separated, they are digested into peptides and deposited on a plate (left), which is then inserted into the mass spectrometer (above). Analysis provides information on the ionized sample’s molecular weight and chemical structure.

**Figure:**
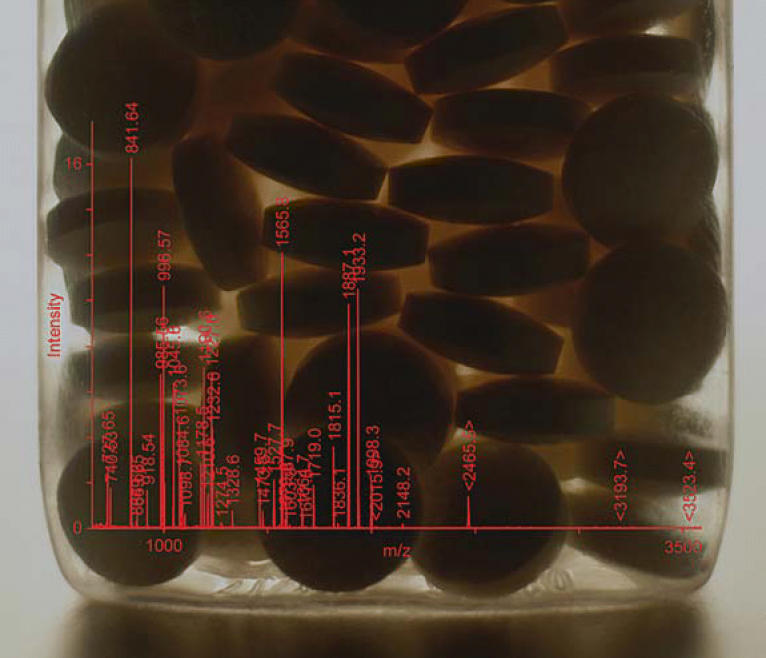
**Peak power.** Spectra like this protein signature generated during acetaminophen studies at the NCT/NIEHS offer clues about the potential toxicity of drugs.

